# Proteomics and Genetic Approaches Elucidate the Circulation of Low Variability *Staphylococcus aureus* Strains on Colombian Dairy Farms

**DOI:** 10.1007/s00248-023-02234-6

**Published:** 2023-05-24

**Authors:** Martha Fabiola Rodríguez, Arlen Patricia Gomez, Claudia Marcela Parra-Giraldo, Andres Ceballos-Garzon

**Affiliations:** 1 School of Health Sciences, Universidad de La Salle, Bogotá, Colombia; 2 School of Veterinary Medicine and Zootechnics, Universidad Nacional de Colombia, Bogotá, Colombia; 3 Proteomics and Human Mycosis Unit, Infectious Diseases Group, Microbiology Department, Pontificia Universidad Javeriana, Bogotá, Colombia; 4 Université Clermont Auvergne, INRAE, MEDiS, 63000 Clermont-Ferrand, France

**Keywords:** Subclinical mastitis, Dairy farm, Environmental, Variability, Milkers, *S. aureus*

## Abstract

*Staphylococcus aureus* is one of the most prevalent pathogens causing bovine mastitis in the world, in part because of its ease of adaptation to various hosts and the environment. This study aimed to determine the prevalence of *S. aureus* in Colombian dairy farms and its relationship with the causal network of subclinical mastitis. From thirteen dairy farms enrolled, 1288 quarter milk samples (QMS) and 330 teat samples were taken from cows with positive (70.1%) and negative California Mastitis Test (CMT). In addition, 126 samples from the milking parlor environment and 40 from workers (nasal) were collected. On each dairy farm, a survey was conducted, and the milking process was monitored on the day of sampling. *S. aureus* was identified in 176 samples, i.e., 138 QMS, 20 from teats, 8 from the milking parlor environment, and 10 from workers’ nasal swabs. Isolates identified as *S. aureus* underwent proteomics (clustering of mass spectrum) and molecular (*tuf*, *coa*, *spa Ig*, *clfA*, and *eno* genes) analysis. Regarding proteomics results, isolates were distributed into three clusters, each with members from all sources and all farms. Concerning molecular analysis, the virulence-related genes *clfA* and *eno* were identified in 41.3% and 37.8% of *S. aureus* isolates, respectively. We provide evidence on the circulation of *S. aureus* strains with limited variability among animals, humans, and the environment. The parameters with the lowest compliance in the farms which may be implicated in the transmission of *S. aureus* are the lack of handwashing and abnormal milk handling.

**Supplementary Information:**

The online version contains supplementary material available at 10.1007/s00248-023-02234-6.

## Introduction

Mastitis is the most common inflammation of the udder in dairy cows [1–4]. This disease has a high worldwide incidence with considerable economic losses and negative implications for hygiene and milk quality [5–7]. Subclinical mastitis is more prevalent than the clinical form, usually precedes it, and lasts longer. Therefore, subclinically affected animals can be the source of infection for other cows in the herd [8]. The appearance of the disease is mainly the result of the interaction between three factors: infectious agents, host, and environmental factors [9]. In this way, the effectiveness in the transmission and permanence of microorganisms (MO) in the herd will depend on population density and factors related to management, such as good milking practices, hygiene, and the type of milking [10, 11]. Although these risk factors have been widely studied, the possibility of finding cows without intramammary infection (IMI) and cows with one or more quarters affected in the same herd suggests that the incidence and prevalence of mastitis also depend on the phenotypic characteristics and genotypic characteristics of MO, and their interaction with the host, with the habitat [12, 13], and with other MO populations, which, in turn, influence the dynamics of these pathogens [14].

According to the transmission mechanisms, origin, and reservoirs, the etiological agents of bovine mastitis are classified as contagious (*Streptococcus agalactiae* and *S. aureus*), opportunistic (non-*aureus* staphylococci (NAS)), and environmental (coliforms and *S. uberis*) [12]. *S. aureus* is the most prevalent contagious pathogen in clinical and subclinical mastitis in the world [15–18]; its transmission occurs mainly during the milking routine since the mammary gland of the adult lactating cow is the most important reservoir of the bacteria, especially in subclinical mastitis. However, *S. aureus* has other extramammary sources, including the skin and mucous membranes of cows, fomites, insects, people, non-bovine animals, soil, and air [19]. Therefore, poor farm management and lack of hygiene represent a high risk for the establishment of this pathogen in the cow and in the herd [20].

*S. aureus* has a large number of virulence factors that allow this bacterium to remain in the udder, facilitating its persistence in dairy herds [21]. The expression of virulence genes appears to depend on the clonal lineage of *S. aureus*. In China, biofilm-forming *S. aureus* clones isolated from milk from cows with subclinical mastitis carried genes encoding aggregation factors (*clfA* and *clfB*), collagen-binding proteins (*cna*), elastin (*ebpS*), laminin (*eno*), fibronectin (*fnbA* and *fnbB*), and fibrinogen (*fib*) [22–24]. The prevalence of the *clfA*, *eno*, *fnbA*, and *fib* genes in isolates of bovine origin is relatively high (60 to 100%) in most countries of the world [22, 25–27]. Additionally, these genes have been related to a high somatic cell (SC) count in milk, indicating that their presence may be related to a greater inflammatory response [28]. In Colombia, it has been found that 81.3% of the isolates of *S. aureus* from IMI are in vitro biofilm formers and carry the *ica* and *bap* genes [29]. Moreover, subclinical mastitis is endemic in the country’s major milk-producing geographical areas, causing large economic losses to producers. The difficulty in its eradication is due in part to the lack of laboratory diagnosis that guides a successful treatment, favoring the persistence of the etiological agents not only in chronically infected animals, but also in the entire herd. Molecular epidemiology studies to identify the microorganisms as well as their possible routes of transmission are needed. Therefore, this study aimed to determine the prevalence of *S. aureus* from dairy farms in the Bogotá Savanna and its relationship with the causation network of subclinical mastitis in the region.

## Materials and Methods

### Farm and Cows

A total of 13 dairy farms located in the Bogotá Savanna, Colombia, were included in the study. Farms that had received orientation on good husbandry and milking practices were selected. All farms had mechanical milking and were classified according to the cattle inventory into small (S: 10–35), medium (M: 36–100), and large (L: > 100) tiers. A total of 330 primiparous or multiparous lactating cows were studied, and cows with clinical manifestations of mastitis or any other systemic or reproductive infection and cows with antimicrobial treatment were excluded.

### Data Collection

On each dairy farm, the owner or administrator provided the data of each farm and the animals. A survey was carried out and the milking process was monitored on the day of sampling. The questionnaire form indicated milking parlor conditions and equipment, as well as compliance with the milking routine, hygiene and biosecurity rules, handling of abnormal milk, staff training, and storage of medicines.

### Sample Collection

In cows, smears were made from the skin of the apex of the teats and quarter milk samples (QMS) were collected according to the standard procedures recommended by the National Mastitis Council (NMC 2014). Briefly, the udders and teats were cleaned using paper towels, and then, the first two streams of foremilk from each quarter were discarded. Each teat apex was scrubbed with cotton moistened with 70% alcohol until it was thoroughly clean. Finally, 10 mL of milk was collected aseptically from each quarter in a sterile falcon tube. In humans, smears were made from the nasal mucosa of the workers. To obtain the environmental samples, smears were made from teat cups, the floor, the walls of the milking parlor, and the tank or canteens. The smears were sampled using swabs and transport medium (Innovation®-Italy). All samples were transported at 4°C to the microbiology laboratory.

### California Mastitis Test (CMT)

The QMS was collected in the wells from a plastic pallet. An equal amount of commercial reagent (sodium dodecyl sulfate 2%) was added to the milk and the paddle was spun to mix the contents. The CMT results were classified according to the concentration scale proposed by Miller and Kearns: *negative (0)* (represents < 200 × 10^3^ cell somatic (CS)/mL), *traces (T)* (200–500 × 10^3^ CS/mL), *mild positive (1)* (400–1500 × 10^3^ CS/mL), *positive (2)* (800–5000 × 10^3^ CS/mL), and *strong positive (3)* (> 5000 × 10^3^ CS/mL). Cows were considered positive for CMT when at least a quarter was positive (≥ T) [30].

### Culture and Microbiological Identification

A total of 1618 samples from 330 cows (QMS: 1288; skin of teats: 330), 40 workers nasal swabs, and 126 smears of environmental samples from the milking parlor were streaked on blood agar and MacConkey agar plates (Difco Laboratories) and incubated at 37°C for 24 to 48 h. For the QMS, 100 mL of milk was streaked on each agar plate. The NMC criteria for the interpretation of bacteriological cultures for the diagnosis of bovine IMI were applied, considering a positive result when a sample presented ≥ 10 UFC/100 mL. For this detection threshold, sensitivity and specificity of 72.0% and 100% have been reported to detect *S. aureus* IMI [31, 32]. Out of 1784 cultures, bacterial growth was found in 905 of them. Colonies were Gram stained and spiked into trypticase soy agar (TSA) (Difco Laboratories) and incubated at 37°C for 18 to 24 h. Isolated colonies were resuspended in sterile saline solution (0.45%) (bioMerieux, Marcy l’Etoile, France) at a concentration of 1.5 × 10^8^ bacteria/mL corresponding to 0.5 McFarland, determined by DensiCHECK plus (BioMerieux, France) and identified using the automated Vitek® 2 compact system (bioMerieux, France).

### Proteomics Identification Using MALDI-TOF MS

Bacteria were also identified by MALDI-TOF MS, according to the Bruker Daltonics protocol. Briefly, one colony from the TSA agar was spotted onto a 96-spot steel plate (Bruker Daltonik, Germany), and allowed to dry at room temperature before the addition of l μL of formic acid and HCCA matrix (provided by the supplier). Each colony was tested in duplicate. Only the spot returning the highest probability score of identification was considered. Protein mass spectra were analyzed using Flex Control® software and MALDI Biotyper version 3.1 7311 reference spectra (main spectra) (Bruker Daltonics, Bremen, Germany). MALDI-TOF MS results were analyzed according to the manufacturer’s technical specifications as follows: correct identification of genus and species (≥ 2.0), correct identification of genus (1.7–2.0), and no reliable identification (< 1.7). The mass spectra from *S. aureus* isolates with a score value of > 2 (140/176) were considered for the preparation of a dendrogram using the respective functionality of the MALDI-TOF MS Biotyper 3.1. The spectra were analyzed as a core-oriented dendrogram using an arbitrary distance level of 1000 as the cut-off.

### Genotypic Identification of *S. aureus*

Among all clades identified in the proteomics analysis, *S. aureus* isolates from different origins were randomly selected for molecular analysis. DNA extraction and purification were carried out using the commercial PureLink™ Genomic DNA Mini Kit (Thermo Fisher Scientific, Waltham, MA), following the manufacturer’s instructions. Subsequently, the *tuf* (Tu elongation factor), *coa* (coagulase), *spa Ig* (binding protein A of the Fc portion of IgG), *clfA* (agglutination factor), and *eno* (laminin-binding protein) genes were amplified by PCR. GoTaq® Green Master Mix (Promega, Madison, USA) was used, reactions were carried out in a final volume of 25 μL, with the following mixture: 12.5 μL of GoTaq® Green Master (2X), 0.5 μL of each primer (0.1 μM), and 2 μL of bacterial DNA (10–30 ng). The primer sequences and PCR conditions are described in Table S1. The PCR products were visualized on a 1.5% agarose gel stained with HydraGreen® (ACTGene, NJ, USA). Gel images were acquired using a gel-documentation system (Thermo Fisher Scientific, USA). Two reference strains (ATCC®) of *S. aureus* positive (43300) and negative (25923) to the mecA-1 gene were used as controls. Additionally, the products of the *tuf*, *coa*, and *spaIg* genes were sequenced in an ABI3730XL sequencer (Applied Biosystems). All sequences were aligned, and the dataset was used to construct a Neighbor-Joining phylogenetic tree using Maximum Composite Likelihood settings by using Molecular Evolutionary Genetics Analysis Version 7 (MEGA7). Evaluation of branch support was performed by Bootstrap statistical analysis with 1000 replicates.

### Repetitive Element PCR Fingerprinting (rep-PCR)

The rep-PCR technique was carried out in a final reaction volume of 50 μL. Each reaction mixture contained 10 μL of 5× Green GoTaq ®Flexi buffer, 3 μL of MgCl2 (25mM), 1 μL of dNTPs (10mM each), a single primer (GTG)—GTGGTGGTGGGTGGTG (14mM), and 0.25 μL GoTaq®DNA polymers (5 U/mL) (Promega, Madison, USA). Subsequently, the chromosomal DNA was added at a concentration of 100 to 200 ng. The reaction mixtures were subjected to 30 cycles consisting of heat denaturation at 94°C for 1 min, primer annealing at 54°C for 1 min, and DNA extension at 72°C for 2 min. Finally, the samples were maintained at 72°C for 5 min for the final extension of DNA. PCR products were visualized on a 1.8% agarose gel stained with HydraGreen® (ACTGene, NJ, USA) at 20 volts for 4 h.

### Statistical Analysis

The data obtained were tabulated and analyzed with descriptive statistics Relative Risk (R.R), and multinominal regression test was used to construct relationships, between categorical variables, and the prevalence of subclinical mastitis estimated with the CMT, and the genus of bacteria and their location identified. For the survey, the percentage of compliance and milking conditions was determined by giving each question a value of 1 for compliance and 0 for non-compliance. The association or dependence of these variables with the CMT score was determined with Pearson’s chi-square test of independence. All statistical analyses were performed considering a significance of *P* < 0.05 using the R language.

## Results

### Animal and Farm Data

Of the 13 farms enrolled, four were from the small tier, five from the medium tier, and four from the large tier. Seven farms had a fixed milking parlor, and six had mobile ones (Table S2). The predominant breed was Holstein, and the CMT was positive (≥ Traces) in 70.1% of cases (CI95% = 64.8–75.1%) (Table S3). The verification of percentage of compliance with good farming practices and their dependence (*χ*^2^) with the CMT score depicted critical points in handling abnormal milk: 8.0% (*χ*^2^: 13.7), staff training, staffing, and cleanliness: 28.2% (*χ*^2^: 18.3), hygiene animal (udder, tails, and flanks): 44.2% (*χ*^2^: 27.5), condition, maintenance, and cleanliness of the milking equipment: 64.1% (*χ*^2^: 20.8), facilities and hygiene of the milking parlor: 77.0% (*χ*^2^: 87.0), and good practices during the milking routine: 77.0% (*χ*^2^: 27.7) (see Fig. 4 in the “Discussion” section).

### Culture and Microbiological Identification

**Fig. 1:**
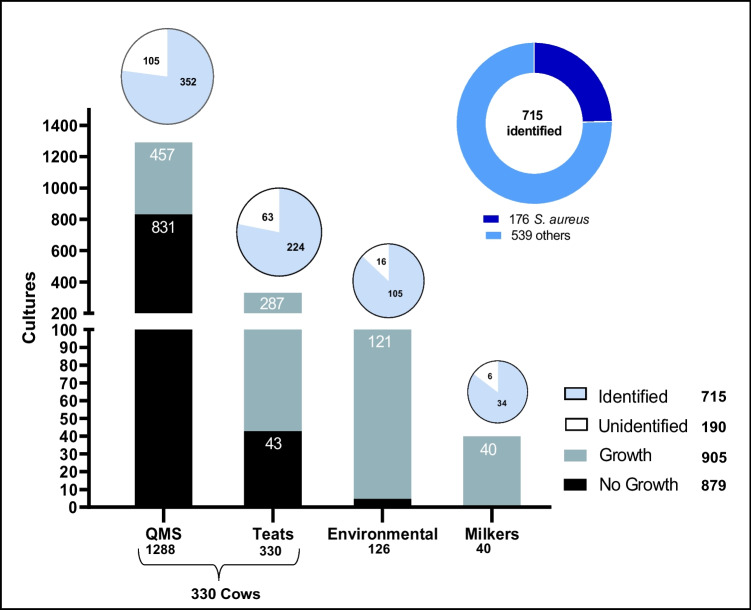
Overall culture and microbial identification results

Of the 1784 cultures taken (QMS: 1288; teats: 330; environmental: 126; milkers: 40), microbial growth was observed in 905 of them (i.e., QMS: 457; teats: 287; environmental: 121; milkers: 40), in which 715 bacteria were identified. Of interest, 176 strains of *S. aureus* were found in the four sources evaluated (i.e., QMS: 138; teats: 20; environmental: 8; milkers: 10) (Fig. 1).

Among the 1288 QMS samples, microbial growth was observed in 457, in which 352 bacteria were identified. The most frequently isolated bacterium was *S. aureus*, found in 138/457 (30.2%) cultures, followed by NAS (*S. chromogenes*, *S. haemolyticus*, *S. warneri*, *S. epidermidis*, *S. hominis*, *S. horserum*, *S. hyicus*, *S. lugdunensis*, and *S. squirrels*) in 74/457 (16.2%) cultures. *Streptococcus* spp. were identified in 66/457 (14.4%), i.e., *S. uberis*, *S. agalactiae*, *S. dysgalactiae*, *S. equi*, and *S. lutetiensis .* Regarding the number of positive CMTs out of the 1288 QMS samples taken, 574/1288 were found to be CMT positive. Interestingly, only in 383/574 (66.7%) microbial growth was seen (Table 1).

**Table 1:** California Mastitis Test and microbial culture result on quarter milk samples

	CMT Negative			CMT positive			Total CMT positive		QMS growth
Grade CMT	0		T	1	2	3			
Culture	*n*	%	*n*	*n*	*n*	*n*	*n*	%	*n*
*S. aureus*	10	(13.5)	19	39	51	19	128	(22.3)	138
NAS	20	(27.0)	11	21	15	7	54	(9.4)	74
*Streptococcus* spp.	8	(10.8)	12	12	18	16	58	(10.1)	66
Other GPC	4	(5.4)	2	9	1	1	13	(2.3)	17
GPB	9	(12.2)	13	5	5	8	31	(5.4)	40
GNB	2	(2.7)	4	8	0	3	15	(2.6)	17
Unidentified	21	(28.4)	15	47	15	7	84	(14.6)	105
Total	**74**	**(100)**	176	192	133	73	**383**	**(100)**	**457**
No growth seen	640		100	51	28	12	191		831
Overall	**714**						**574**		**1288**

In bold, the total values for each condition are shown

*T*, traces; *GPC*, gram-positive cocci; *GPB*, gram-positive bacilli; *GNB*, gram-negative bacilli

On teat apex skin, 330 cultures were taken, and microbial growth was observed in 287 of them. NAS was found in 119 out of 287 (41.5%), of which *S. haemolyticus* (21.8%), *S. warneri* (16.8%), *S. chromogenes*, *S. hominis* (14.3%), and *S. epidermidis* (5.0%) were the most frequent species. *S. aureus* was identified in 20/287 samples (7.0%) (Table S4). From the environment of the farms’ milking parlor, 126 samples were taken. Gram-negative bacilli (GNB) were found in 39/121 cultures (32.2%) and NAS 28/121 (23.1%). *S. aureus* was identified in 8/121 (6.6%) (Table S5). Finally, in cultures from milkers, 10/40 (25.0%) were identified as *S. aureus*, 10/40 as *S. epidermidis*, 7/40 (17.5%) as NAS (*S. auricularis*, *S. lentus*, *S. haemolyticus*, and *S. warneri*), and 7/40 (17.5%) as GNB, mainly *Enterobacteriaceae*.

Overall, of the 715 bacteria identified, *S. aureus*, *S. chromogenes*, *S. warneri*, and *S. haemolyticus* were the most frequently isolated species. However, *S. aureus* (24.6%) was the most frequent, both in the totality of the farms and in each group analyzed (small tier: 15%; medium tier: 17%; large tier: 34%), being significant (*P* < 0.05) the predominance of *S. aureus* in large tier farms (Table S6).

### Identification and Analysis by MALDI-TOF MS

**Fig. 2:**
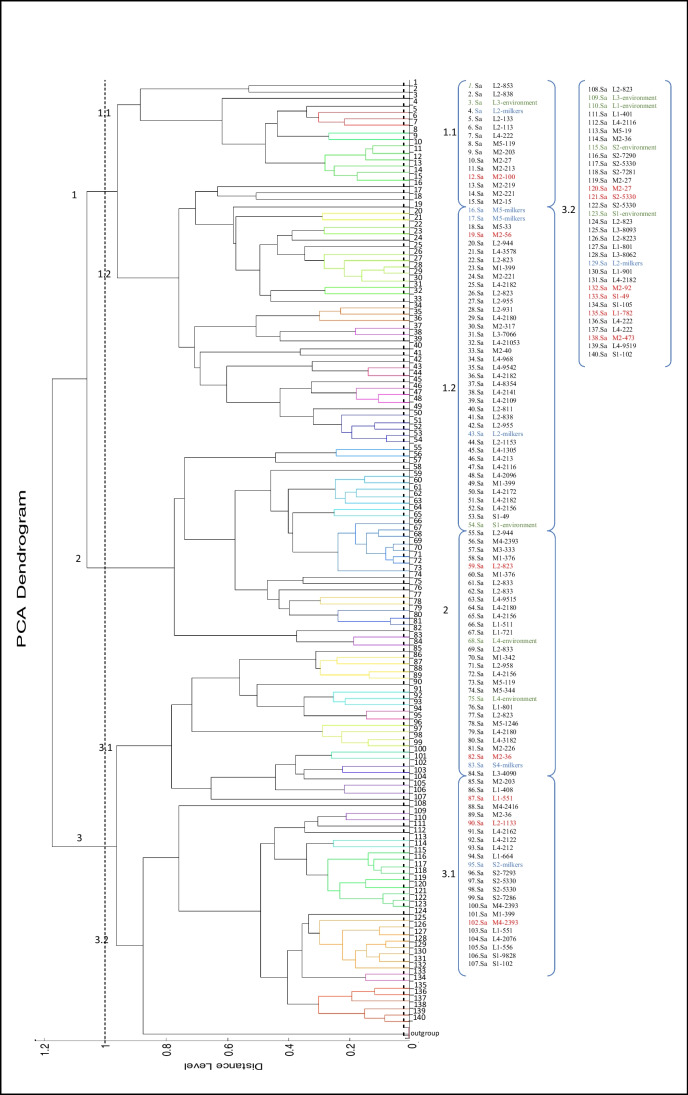
Dendrogram of *S. aureus* (Sa) isolates from quarter milk samples (list in black) and teats from cows (list in red), from milkers (list in blue), and from the environment (list in green). Cut-off point at a distance level of 1. Each sample indicates the tier of the farm (L: large, M: medium, S: small) followed by the identification code of the farm and cow

Of the 715 bacteria identified, 176 strains comprised *S. aureus*, of which 140 were included in the phylogenetic analysis (MALDI-TOF score > 2), all taxonomically identified with NCBI numbers 1280 and 46170. *S. aureus* isolates were distributed in three clades regardless of their origin: milk and skin of the teats of cows, environment, and humans. The strains from the large farms were distributed across the three clades, as were those from two medium-sized farms (M2 and M5). In clade 1, 54 isolates from six farms were distributed in two subgroups and 15 clusters. The clusters with more than four isolates were from different farms and sources, except for the cluster of position 10–15 where milk and teat isolates from the same farm were found (Fig. 2).

Clade 2 was the smallest, with 30 isolates from nine farms, from all sources arranged in four clusters. Clade 3 grouped 56 isolates from 10 farms distributed in 13 clusters. In subgroup 3.2, the largest number of isolates from the environment (4) was found. In addition, six isolates from the microbiota of the teats, 22 from milk, and one human isolate (position 129) were located, which were obtained from all the large farms, (4) medium, and (2) small, of which one (S2) was only located in this clade. The remaining human isolates were found singly (position 16) or in a two-member cluster (position 14, 17, 43, 83, or 95), in which they were always accompanied by milk isolates from the same or different farms. The environmental isolates were found in clusters with isolates of animal origin and from different farms, except isolates 3 and 109, which did not form clusters.

### Genotypic Identification of *S. aureus*

**Fig. 3:**
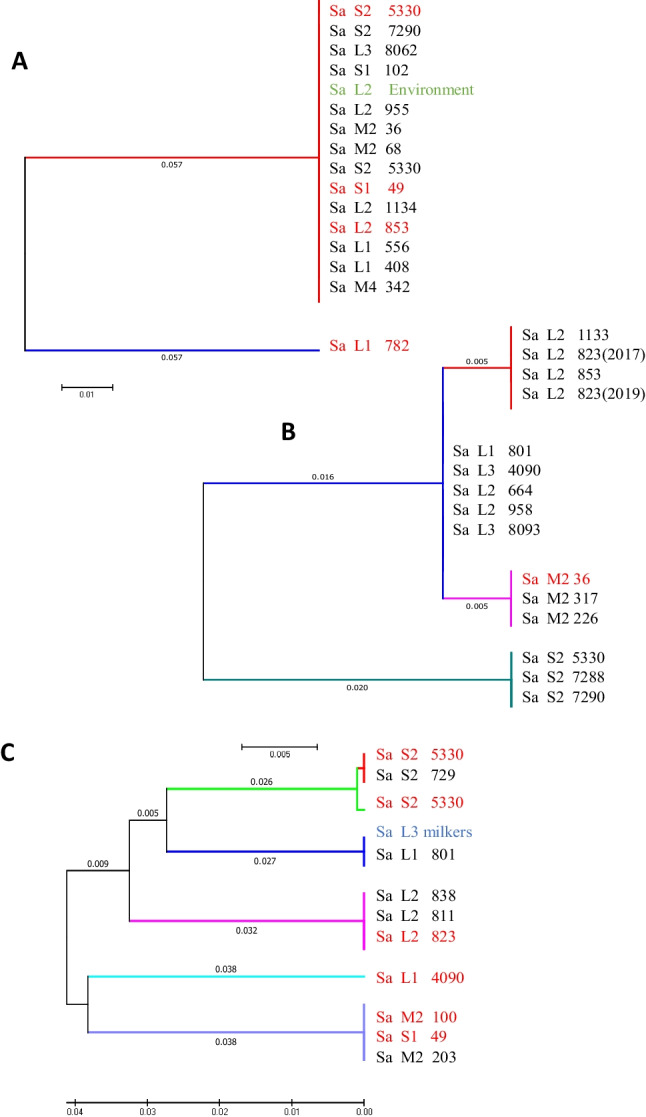
Phylogenetic relationships of randomly selected *S. aureus* isolates from all dairy farms enrolled, through the analysis of spa Ig (**A**), coa (**B**), and tuf (**C**) genes. Isolates from QMS (black) and skin of the teats (red) from cows, from milkers (blue), and from the environment (green). Each sample indicates the tier of the farm (L: large, M: medium, S: small) followed by the identification code of the farm and cow

PCR amplification and sequencing of the variable region of the virulence genes *spa Ig* and *coa* allowed the identification of two and four clades, respectively, and the sequencing of the *tuf* gene, widely used for the phenotypic identification of the *S. aureus* species, also revealed two clades. This assay confirms the predominance of a few clades, with several clusters, some specific to each farm, as observed in the proteomics analysis, but most with isolates from different farms that share a similarity among them. Likewise, consistent with the first dendrogram obtained from the proteomics analysis, each clade contains *S. aureus* from all sources, especially QMS and teat isolates, from the different farms in the region (Fig. 3). The study of genes encoding virulence factors, *clfA* and *eno*, showed that a high percentage (41.3%) of *S. aureus* circulating in the region have the *cflA* gene, especially the isolates from teats and milk. Furthermore, the *eno* gene was found in 37.8% of isolates, mainly in *S. aureus* from the environment and from the milk of cows with subclinical mastitis (Table 2).

**Table 2:** Percentage of virulence genes found in randomly selected *S. aureus* isolates from different sources: quarter milk samples (QMS), teats, environment, and milkers

*Source*	*spa Ig*	*coa*	*cflA*	*eno*
	*n* (%)	*n* (%)	*n* (%)	*n* (%)
QMS (138)	105/108 (97.2%)	117/117 (100.0%)	46/112 (41.1%)	45/112 (40.2%)
Teats (20)	12/15 (80.0%)	18/18 (100.0%)	9/16 (56.3%)	4/16 (25.0%)
Milkers (10)	8/8 (100.0%)	10/10 (100.0%)	3/8 (37.5%)	2/8 (25.0%)
Environment (8)	5/6 (83.3%)	7/7 (100.0%)	1/7 (14.3%)	3/7(42.9%)
Total	**130/137 (95.0%)**	**152/152 (100.0%)**	**59/143 (41.3%)**	**54/143 (37.8%)**

In bold, the total values for each condition are shown

The study of genetic variability by means of the rep-PCR technique of *S. aureus* showed variability in the banding patterns of isolates in each farm and among them. However, genotypes with the same banding pattern were also observed in the milk of cows with subclinical mastitis and the environment in farms S2 and M2, similar to what was observed by the proteomics and molecular analysis, which showed a high similarity between isolates from these farms (Fig. S1). This confirms the circulation of the close-genetic variants between animals, humans, and the environment within the farms and among the farms of the Bogotá Savanna.

## Discussion

The detection of predominant genotypes and phenotypes in the milk of infected cows from different herds suggests that certain strains are more effective in causing mastitis and in spreading among animals in the same herd [33]. In this study, the prevalence of subclinical mastitis reached 70.1% (CI95 = 64.8–75.1%), being higher than the previous prevalence reported (55.2%) in the same region [16], and other geographical areas, in Colombia, i.e., 11.3% have been reported in the department of Cordoba [34], 37.2% in Antioquia [35], and 39.8% in the Boyacá high plateau [36].

In subclinical mastitis, diagnosis is challenging since it depends on a confirmatory test [37]. However, the SC count is an indirect measure of the infectious process, which is easily and inexpensively performed both in the laboratory and under field [38].

The increase in the prevalence of subclinical mastitis in the region could be explained by the lack of management of the identified main risk factors. In the milking routine, the major deficiency found was the poor cleaning of udders, flanks, and tails of the animals, and in addition, the lack of washing of the milkers’ hands and forearms before starting the milking routine, which is significantly associated with CMT positivity. In small-scale dairy production systems in developing countries, the high prevalence of bovine mastitis due to contagious and environmental pathogens is associated with poor hygiene in the milking process [8], and the lack of cleanliness of the cows, respectively [10]. Consistent with previous reports in the region [36, 39, 40], *S. aureus* was the most prevalent etiologic agent in cows with subclinical mastitis. Its ability to survive in the environment and infect various hosts allows it to be transmitted by multiple routes [19]. Thus, poor farm management and lack of hygiene represent a high risk for the establishment of this pathogen in the cow and on the farm [20].

**Fig. 4:**
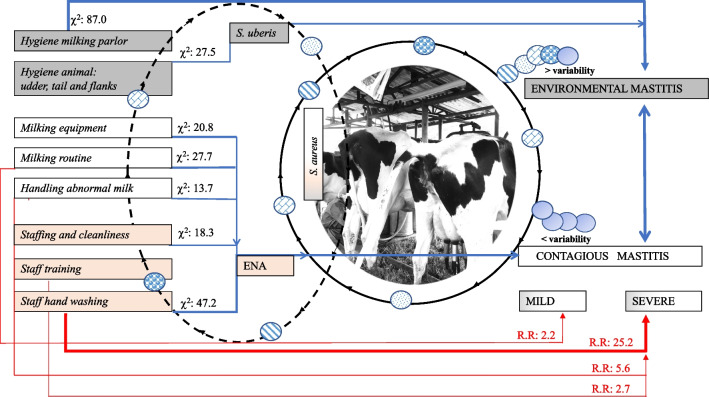
Graphic representation of the circulation of *S. aureus* in dairy farms of the Bogotá Savanna. The blue arrows indicate the dependence between the variables and the presence of mastitis (*P* < 0.05) (Pearson’s chi-square test of independence). The red arrows denote the Relative Risk (R.R.) generated by the variable and the presence of mild or severe mastitis (*P* < 0.05) (multinomial regression test). The black arrows represent the circulation of different strains of *S. aureus* permanently or intermittently between the environment, humans, and animals

Herein, we observed the circulation of clustered strains of *S. aureus* among animals, humans, and the environment. The lack of hand washing represented the most important risk factor (R.R: 25.2) associated with the prevalence of subclinical mastitis. Other risk factors were poor handling of abnormal milk (R.R: 5.6), lack of milker training (R.R: 2.7), and failures in the milking routine (R.R: 2.2) (Fig. 4). Conversely, disinfection and sealing of the teats during milking, as well as dry cow therapy, were the parameters with the highest compliance. This suggests that some strains of *S. aureus* that circulate in the region could have environmental reservoirs that guarantee their long-term persistence. Interestingly, an African study in smallholder dairy farms reported strains of *S. aureus* adapted to the environment and associated with mastitis without the predominance of a particular variant [15]. Studies have also shown that in dairy production systems in developing countries, where mastitis-infected animals are often not slaughtered, the usual source of IMI is udders infected with the pathogen [15, 17]. If the lack of animal hygiene and good milking practices by workers is added to this situation, pathogens from the surrounding environment become another important source of mastitis that is dynamically transmitted between humans, animals, and the environment of the farms [2, 17].

On the other hand, the environmental strains found in this study showed a high prevalence of the *eno* virulence gene, similar to the prevalence found in milk isolates from cows with subclinical mastitis. This gene codes for laminin-binding protein, one of the main components of the basement membrane of the mammary epithelium. Their acquisition by environmental strains will facilitate adherence and the ability to invade and spread to other hosts. The *cflA* gene was amplified in most human and teat *S. aureus* strains, which together with the *clfB* gene are highly prevalent in bovine *S. aureus* strains worldwide [22, 25–27], indicating that they segregate clonally among bacteria isolated from bovines. These genes code for aggregation and adhesion proteins, necessary for the adherence of the bacteria to the mammary epithelium, which possibly explains why it was found mainly in *S. aureus* strains isolated from the skin of the teats.

The main limitation of this study is the number of farms enrolled which may not reflect the situation in the entire region. Whether dairy cows with bovine mastitis can become possible reservoirs of bacteria associated with humans, and whether humans are responsible for the transmission and persistence of *S. aureus* strains in animals and the environment are hypotheses that require further investigation from a One Health perspective to improve animal welfare and food quality and thus promote human health.

In conclusion, regardless of the source of origin, *S. aureus* was identified as the most frequently encountered microorganism in a dairy environment of the Bogotá Savanna. The prevalence of subclinical mastitis reached 70.1%. Furthermore, based on proteomics and genetic analysis, low diversity among circulating clones was observed. Of interest, 37.8% and 41.3% of the strains from teats and the environment harbored genes (*eno & cflA*) associated with adherence and the ability to invade and spread to other hosts, respectively. The lack of hand washing represented the most important risk factor associated with the prevalence of subclinical mastitis in the region.

### Supplementary Information

ESM 1 (DOCX 240 kb)
