# Supplementary material for: Proteomics and Genetic Approaches Elucidate the Circulation of Low Variability Staphylococcus aureus Strains on Colombian Dairy Farms
Source: Microb Ecol. 2023 May 24;86(4):2320–9. doi: 10.1007/s00248-023-02234-6 (PMC10640432; doi:10.1007/s00248-023-02234-6)
Supplement: Supplementary file 1 — (DOCX 240 kb) [file 248_2023_2234_MOESM1_ESM.docx]

**Table S1.** List of primers used for detection of identification and virulence genes in *Staphylococcus aureus* isolates from dairy farms.

| **Gene** | ***Primers sequence (5`- 3`)*** | **Product size (pb)** | **Program** | **Reference** |
| --- | --- | --- | --- | --- |
| ***Tuf*** | 5´-GCCAGTTGAGGACGTATTCT-3´ 5´-CCATTTCAGTACCTTCTGGTAA-3´ | 412 | 1 | (Hwang et al. 2011) |
| ***coa*** | 5´-ATAGAGATGCTGGTACAGG-3´ 5´-GCTTCCGATTGTTCGATGC-3´ | 627, 710, 910 | 2 | (Kalorey et al. 2007) |
| ***spaIg*** | 5´-CACCTGCTGCAAATGCTGCG-3´ 5´-GGCTTGTTGTTGTCTTCCTC-3´ | 590, 810, 970 | 3 | (Kalorey et al. 2007) |
| ***clfA*** | 5´-ATTGGCGTGGCTTCAGTGCT-3´ 5´-CGTTTCTTCCGTAGTTGCATTTG-3´ | 292 | 4 | (Tristan et al. 2003) |
| ***Eno*** | 5´-ACGTGCAGCAGCTGACT-3´ 5´-CAACAGCATCTTCAGTACCTTC-3´ | 302 | 5 | (Simojoki et al. 2012) |

***1.*** *95°C for 15 min, then 35 cycles of 95°C for 30 s, 56°C for 30 s, and 72°C for 45 s.*

***2.*** *94°C for 5 min, then 30 cycles of 94°C for 40 s, 58°C for 60 s, 72°C for 60 s.*

***3.*** *94°C for 5 min, then 35 cycles of 94°C for 60 s, 57°C for 60 s, 72°C for 60 s.*

***4.*** *94°C for 5 min, then 25 cycles of 94°C for 1 min, 55°C for 1 min, 72°C for 1 min.*

***5.*** *94°C for 5 min, then 30 cycles of 95°C for 30 s, 54°C for 30 s, 72°C for 30 s.*

*The final extension for all programs was 72°C for 10 min.*

**Table S2.** Description and Classification of the dairy farms of Bogotá savanna included in this study.

| **Tier of farm** | **Farm´s code** |  | | **Area  (ha)** | **Grazing** | **Race** | **Cows** | **Milking cows** | **Milking parlor** |
| --- | --- | --- | --- | --- | --- | --- | --- | --- | --- |
|  |  | **City** | **Village** |  |  |  |  |  |  |
| **Small:  10-35 cows** | **P1** | Nemocón | Casa Blanca | 3.6 | Stripe | *Holstein* | 25 | 21 | Fixed |
|  | **P2** | Sopo. | San Gabriel | 8 | Rotational | *Holstein* | 21 | 16 | Mobile |
|  | **P3** | Zipaquirá. | Rio Frio | 6.4 | Rotational | *Holstein* | 31 | 16 | Mobile |
|  | **P4** | Calera | San Cayetano | 2 | Stripe | *Holstein, Swedish Red, Ayrshire* | 20 | 17 | Fixed |
| **Medium: 36-100 cows** | **M1** | Suesca | Palmira | 30 | Rotational | *Holstein* | 73 | 42 | Fixed |
|  | **M2** | Sopo. | Aposentos | 20 | Rotational | *Holstein* | 41 | 34 | Mobile |
|  | **M3** | Zipaquirá. | Rio Frio | 20 |  | *Ayrshire* | 49 | 28 | Mobile |
|  | **M4** | Cogua. | Rodamontal | 20 | Rotational | *Holstein* | 42 | 37 | Mobile |
|  | **M5** | Sopo. | Aposentos | 24 | Stripe | *Holstein, Jersey* | 60 | 38 | Fixed |
| **Large: >100cows** | **G1** | Nemocón. | Checua | 41 | Rotational | *Ayrshire* | 131 | 64 | Fixed |
|  | **G2** | Choconta | Chinita | 260 | Rotational | *Holstein* | 185 | 68 | Mobile |
|  | **G3** | Sopo | Aposentos | 80.3 | Rotational | *Holstein, Swedish Red* | 173 | 35 | Fixed |
|  | **G4** | Choconta | Aucio | 67 | Rotational | *Holstein, Normand* | 104 | 60 | Fixed |

**Table S3.** Results of the California Mastitis Test by farm tier.

| **Tier of farm** | **Farm´s code** | **Cows tested** | **CMT  % Positive** (CI:95%)* | **CMT  % Positive** |
| --- | --- | --- | --- | --- |
|  |  | *n* |  | (CI:95%) |
| **Small** | P1 | 21 | **66.7** (43**.**1-84**.**5) | **72.2** (45**.**3-89.5) |
|  | P2 | 16 | **81.3** (53**.**7-95**.**0) |  |
|  | P3 | 12 | **58.3** (28**.**6-83,1) |  |
|  | P4 | 17 | **82.4** (55**.**8-95**.**3) |  |
| **Medium** | M1 | 19 | **52.6** (29**.**5-74,8) | **63.7** (42**.**8-80**.**9) |
|  | M2 | 27 | **59.2** (39**.**0-76,9) |  |
|  | M3 | 27 | **66.7** (46**.**0-82,8) |  |
|  | M4 | 27 | **63.6** (40**.**8-81**.**9) |  |
|  | M5 | 38 | **76.3** (59**.**4-87**.**9) |  |
| **Large** | G1 | 32 | **75.0** (56**.**3-87**.**9) | **74.6** (56**.**1-87**.**0) |
|  | G2 | 36 | **58.3** (40**.**9-74**.**0) |  |
|  | G3 | 28 | **75.0** (54**.**8-88**.**6) |  |
|  | G4 | 30 | **90.0** (72**.**3-97**.**4) |  |
| **Total** |  | **330** | **70.1 (64.8-75.1)** |  |

*** CI*:* *Confidence interval*

**Table S4.** Bacterial culture results on skin of the teats of milking cows

| **Microbiological cultures** | | **n** | **%** |
| --- | --- | --- | --- |
| **Unidentified** |  | 63 | (22.0) |
| **NAS** | *S. haemolyticus, S. warneri, S. chromogenes, S. hominis, S. epidermidis, S. arlettae, S. auricularis, S. capit~~i~~s, S. schleiferi, S. equorum, S. hyicus, S. lentus, S. lugdunensi, S. simiae, S. simulans, S. vitulinus, y S. xylosus* | 119 | (41.5) |
| ***S. aureus*** |  | 20 | (7.0) |
| ***S. uberis*** |  | 4 | (1.4) |
| ***Streptococcus thoraltensis*** |  | 1 | (0.3) |
| ***Enterococcus*** |  | 1 | (0.3) |
| **Others GPC** | *Aerococcus viridans, Kocuria spp, Lactococcus lactis, Leuconostoc mesenteroides, Micrococcus luteus, Rothia terrae* | 27 | (9.4) |
| ***Corynebacterium spp*** |  | 2 | (0.7) |
| **Others GPB** | *Arthrobacter gandavensis, Bacillus spp, Lactobacillus acidophilus* | 30 | (10.5) |
| **GNB** | *Klebsiella oxytica, Pantoea agglomerans, Pasterella canis, Serratia ficaria, Sphingomona paucimobilis, Acinetobacter lwoffii y Pseudomona luteola* | 20 | (7.0) |
| **Total** |  | **287** | **100** |
| No growth seen |  | 43 |  |
| **Overall** |  | **330** |  |

*NAS:* non-*aureus* staphylococci *GPC: Gram Positive Cocci, GPB: Gram Positive Bacilli,*

*GNB: Gram Negative Bacilli*

**Table S5.** Bacterial culture results from environmental samples

| **Microbiological cultures** | **Teat cups** | **Milking parlor** | **Teat sealant cups** | **Tank or canteens** | **Water** | **Total** | |
| --- | --- | --- | --- | --- | --- | --- | --- |
|  | **n** | **n** | **n** | **n** | **n** | **n** | **%** |
| **Unidentified** | 4 | 3 | 1 | 2 | 6 | 16 | (13.2) |
| **GNB** *Morganella morganii, Pseudomona spp y Pantoea spp, Hafnia alvei, Acinetobacter spp, Aeromonas hydrophila, Enterobacter spp, E. coli, Sphingomona paucimobilis, Klebsiella spp y Serratia spp* | 15 | 7 | 4 | 8 | 5 | 39 | (32.2) |
| **NAS** *S. chromogenes, S. equorum, S. haemolyticus, S. vitilinus y S. xylosus, S. epidermidis, S. hominis, S. lentus, S. saprophyticus, S. simulans, S. warneri* | 20 | 6 | 1 | 1 | 0 | 28 | (23.1) |
| *S. uberis* | 3 | 0 | 0 | 0 | 0 | 3 | (2.5) |
| *S. aureus* | 7 | 1 | 0 | 0 | 0 | 8 | (6.6) |
| **Others GPC** *Aerococcus viridans, Kocuria spp, Rothia terrae, Enterococcus spp, Leuconostoc mesenteroides, Lactococcus spp* | 12 | 5 | 0 | 6 | 0 | 23 | (19.0) |
| **GPB** | 3 | 0 | 1 | 0 | 0 | 4 | (3.3) |
| **Total** | **64** | **22** | **7** | **17** | **11** | **121** | **(100)** |
| No growth seen |  |  |  |  |  | 5 |  |
| **Overall** |  |  |  |  |  | **126** |  |

**Table S6.** Percentage of the bacteria identified in each farm tier.

|  | **Large tier** | | **Medium tier** | | **Small tier** | | **Total** | |
| --- | --- | --- | --- | --- | --- | --- | --- | --- |
| **Bacteria** | **n** | **%** | **n** | **%** | **n** | **%** | **n** | **%** |
| *S. aureus* | 110 | (34.4) | 44 | (17.3) | 22 | (15.4) | 176 | (24.6) |
| *S. chromogenes* | 24 | (7.5) | 5 | (2.0) | 16 | (11.2) | 45 | (6.3) |
| *S. warneri* | 22 | (6.9) | 19 | (7.5) | 3 | (2.1) | 44 | (6.2) |
| *S. haemolyticus* | 18 | (5.6) | 14 | (5.5) | 11 | (7.7) | 43 | (6.0) |
| *S. epidermidis* | 7 | (2.2) | 11 | (4.3) | 10 | (7.0) | 28 | (3.9) |
| *S. hominis* | 7 | (2.2) | 14 | (5.5) | 5 | (3.5) | 26 | (3.6) |
| Others ENA | 34 | (10.6) | 11 | (4.3) | 9 | (6.3) | 54 | (7.6) |
| *S. uberis* | 20 | (6.3) | 28 | (11.0) | 11 | (7.7) | 59 | (8.3) |
| *Streptococcus spp* | 5 | (1.6) | 7 | (2.7) | 3 | (2.1) | 15 | (2.1) |
| Others GPC | 24 | (7.5) | 28 | (11.0) | 18 | (12.6) | 67 | (9.4) |
| *Corynebacterium spp* | 7 | (2.2) | 9 | (3.5) | 5 | (3.5) | 21 | (2.9) |
| Others GPB | 16 | (5.0) | 28 | (11.0) | 13 | (9.1) | 57 | (8.0) |
| *Enterobacteriacea* | 12 | (3.8) | 22 | (8.6) | 5 | (3.5) | 39 | (5.5) |
| Others GNB | 14 | (4.4) | 15 | (5.9) | 12 | (8.4) | 41 | (5.7) |
| **Total** | **320** | (**100)** | **255** | (**100)** | **143** | (**100)** | **715** | (100) |

**Fig S1.** Rep-PCR of *S. aureus* isolated from QMS and teats from cows, milkers and the environment from dairy farms in the Bogotá savanna. L_1_-_2_: l3-QMS, L_3_: l3-milkers, L_4-5_: l3-environment, L_6_: m4-QMS, L_7-9_: m1-QMS, teat, QMS, L_10_: Ladder, L_11-16_: l2-QMS, L_17_: l2-teat, L_18_: l2-QMS, L_19_: s1-environment, L_20-22_: m2-QMS, L_23_: *S. aureus* ATCC 43000, L_24_: *S. aureus* ATCC 25925. Each line indicates the tier of the farm (l: large, m: medium, s: small) followed by the sample types.
